# A New Giant Titanosauria (Dinosauria: Sauropoda) from the Late Cretaceous Bauru Group, Brazil

**DOI:** 10.1371/journal.pone.0163373

**Published:** 2016-10-05

**Authors:** Kamila L. N. Bandeira, Felipe Medeiros Simbras, Elaine Batista Machado, Diogenes de Almeida Campos, Gustavo R. Oliveira, Alexander W. A. Kellner

**Affiliations:** 1 Laboratório de Sistemática e Tafonomia de Vertebrados Fósseis, Departamento de Geologia e Paleontologia, Museu Nacional/Universidade Federal do Rio de Janeiro. Quinta da Boa Vista s/n, 20940-040, São Cristóvão, Rio de Janeiro, RJ, Brazil; 2 Petróleo Brasileiro S.A. (PETROBRAS), Avenida República do Chile, 330, -17° andar, Centro, Rio de Janeiro, RJ, Brazil; 3 Universidade Estácio de Sá, Rua André Rocha, 838, Taquara, 22710-560, Rio de Janeiro, RJ, Brazil; 4 Museu de Ciências da Terra, CPRM. Av. Pasteur, 404, Urca, 22290-240, Rio de Janeiro, RJ, Brazil; 5 Universidade Federal Rural de Pernambuco, Departamento de Biologia. Rua Dom Manuel de Medeiros s/n, Dois Irmãos, 52171-900, Recife, PE, Brazil; State Museum of Natural History, GERMANY

## Abstract

Titanosaurian dinosaurs include some of the largest land-living animals that ever existed, and most were discovered in Cretaceous deposits of Argentina. Here we describe the first Brazilian gigantic titanosaur, *Austroposeidon magnificus* gen. et sp. nov., from the Late Cretaceous Presidente Prudente Formation (Bauru Group, Paraná Basin), São Paulo State, southeast Brazil. The size of this animal is estimated around 25 meters. It consists of a partial vertebral column composed by the last two cervical and the first dorsal vertebrae, all fairly complete and incomplete portions of at least one sacral and seven dorsal elements. The new species displays four autapomorphies: robust and tall centropostzygapophyseal laminae (cpol) in the last cervical vertebrae; last cervical vertebra bearing the posterior centrodiapophyseal lamina (pcdl) bifurcated; first dorsal vertebra with the anterior and posterior centrodiapophyseal laminae (acdl/pcdl) curved ventrolaterally, and the diapophysis reaching the dorsal margin of the centrum; posterior dorsal vertebra bearing forked spinoprezygapophyseal laminae (sprl). The phylogenetic analysis presented here reveals that *Austroposeidon magnificus* is the sister group of the Lognkosauria. CT scans reveal some new osteological internal features in the cervical vertebrae such as the intercalation of dense growth rings with camellae, reported for the first time in sauropods. The new taxon further shows that giant titanosaurs were also present in Brazil during the Late Cretaceous and provides new information about the evolution and internal osteological structures in the vertebrae of the Titanosauria clade.

## Introduction

Titanosaurs are considered a cosmopolitan group of dinosaurs and represent one of the most abundant and diverse clade within Sauropoda [1, 2]. The record of those dinosaurs has increased greatly in recent decades, particularly in South America due to discoveries made in Argentina and Brazil (e.g., [3–8]). Several specimens have been reported from India [9, 10], Africa [11, 12, 13, 14], Australia [15], and recently also from Antarctica [16]. Although regarded by some as typical Gondwanan taxa [17, 18], titanosaurs have also been reported in Laurasian continents (e.g., [19–25]), albeit in lower diversity.

Regarding South America, Brazil and Argentina show a similar dinosaur fauna during the Late Cretaceous [26, 27]. Up to date, there are nine recognized titanosaur species from Brazil, one from the Early Cretaceous Areado Group (Sanfranciscana Basin; *Tapuiasaurus macedoi* Zaher *et al*., 2011) and eight from the Late Cretaceous Bauru Group (Paraná Basin; *Gondwanatitan faustoi* Kellner & Azevedo, 1999; *Trigonosaurus pricei* Campos *et al*., 2005; *Baurutitan britoi* Kellner *et al*., 2005; *Maxakalisaurus topai* Kellner *et al*., 2006; *Adamantisaurus mezzalirai* Santucci and Bertini, 2006; *Uberabatitan ribeiroi* Salgado and Carvalho, 2008; *“Aeolosaurus” maximus* Santucci and Arruda-Campos, 2011, and *Brasilotitan nemophagus* Machado *et al*., 2013).

**Fig 1:**
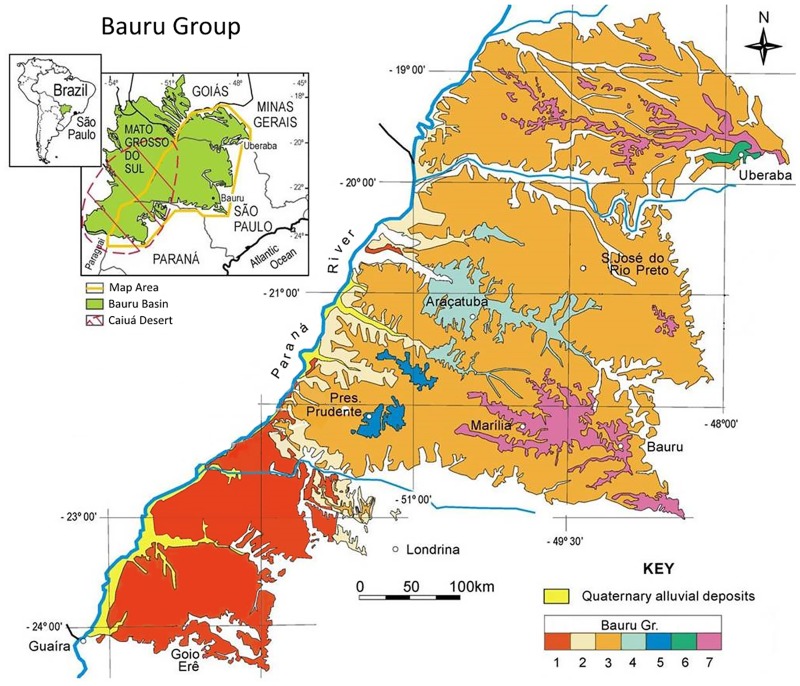
Map showing the location of the Bauru Group (modified from Fernandes *et al*., 2007). Geological map showing the locations of the outcrops of the Bauru Group in southwestern São Paulo state regarding the following formations: (1) Cauiá, (2) Santo Anastácio, (3) Adamantina, (4) Araçatuba, (5) Presidente Prudente, (6) Uberaba and (7) Marília.

Here we describe a new sauropod dinosaur, *Austroposeidon magnificus* gen. et sp. nov., also from the Bauru Group, more specifically from the Presidente Prudente Formation [28, 29, 30, 31] (Fig 1). The specimen consists of cervical and dorsal vertebrae that were collected by Llewellyn Ivor Price in 1953 at the outskirts of the Presidente Prudente City, southwestern São Paulo State [28], and is housed at the Museu de Ciências da Terra (MCT; Companhia de Pesquisa de Recursos Minerais of Rio de Janeiro—CPRM).

Besides dinosaurs, the Cretaceous deposits in the region of Presidente Prudente have provided many vertebrate fossils such as fishes, turtles, crocodyliforms and even squamates [32, 33, 34, 35].

## Materials and Methods

### Anatomical Nomenclature and Abbreviations

We employed the Romerian terminology and the directional terms instead of veterinarian alternatives [36] and followed recent recommendations regarding the identification of vertebral laminae [37, 38] and fossae [39]. ‘‘Anterior” and ‘‘posterior,” for example, are used as directional terms rather than the veterinarian alternatives ‘‘rostral” or ‘‘cranial” and ‘‘caudal”. The abbreviations are as follows:

Anatomical abbreviations: **c**, centrum; **d**, diapophysis; **mec**, medial crest; **nc**, neural channel; **pa**, parapophysis; **pl**, pleurocoel; **poz**, postzygapophysis; **prz**, prezygapophysis; **s**, neural spine; **st**, spine tubercle.

Laminae: **acdl**, anterior centrodiapophyseal lamina; **acpl**, anterior centroparapophyseal lamina; **cpol**, centropostzygapophyseal lamina; **cprl**, centroprezygapophyseal lamina; **pcdl**, posterior centrodiapophyseal lamina; **pcpl**, posterior centroparapophyseal lamina; **podl**, postzygodiapophyseal lamina; **posl**, postspinal lamina; **ppdl**, paradiapophyseal lamina; **prdl**, prezygodiapophyseal lamina; **prsl**, prespinal lamina; **spdl**, spinodiapophyseal lamina; **spol**, spinopostzygapophyseal lamina; **sprl**, spinoprezygapophyseal lamina; **spll**, suprapleurocoel lamina (new); **tprl**, intraprezygapophyseal lamina; **tpol**, intrapostzygapophyseal lamina.

Fossae: **cdf**, centrodiapophyseal fossa; **cpof**, centropostzygapophyseal fossa; **pacdf**, parapophyseal centrodiapophyseal fossa; **pacprf**, parapophyseal centroprezygapophyseal fossa; **pocdf**, postzygapophyseal centrodiapophyseal fossa; **posdf**, postzygapophyseal spinodiapophyseal fossa; **prcdf**, prezygapophyseal centrodiapophyseal fossa; **prpadf**, prezygapophyseal paradiapophyseal fossa; **prsdf**, prezygapophyseal spinodiapophyseal fossa; **sdf**, spinodiapophyseal fossa; **spof**, spinopostzygapophyseal fossa; **splaf**, suprapleurocoel accessory fossa (new).

### Taxa Compared

The new species, *Austroposeidon magnificus* gen. et nov. sp., is compared with the following titanosaurs: *“Aeolosaurus” maximus* Santucci and Arruda-Campos, 2011; *Andesaurus delgadoi* Calvo and Bonaparte, 1991; *Alamosaurus sanjuanensis* Gilmore, 1922; *Ampelosaurus atacis* LeLoeuff, 1995; *Argentinosaurus huinculensis* Bonaparte and Coria, 1993; *Atsinganosaurus velauciensis* Garcia *et al*., 2010; *Brasilotitan nemophagus*; *Bonitasaura salgadoi* Apesteguía, 2004; *Elaltitan lilloi* Mannion and Otero, 2012; *Epachthosaurus sciuttoi* Powell, 1990; *Futalognkosaurs dukei* Calvo *et al*. 2007a; *Gondwanatitan faustoi*; *Isisaurus colberti* (Jain and Bandyopadhyay, 1997); *Ligabuesaurus leanzai* Bonaparte *et al*., 2006; MCT 1487-R (known as “Série A” by Powell, 1987); *Malawisaurus dixeyi* Jacobs *et al*., 1993 (Gomani, 2005); *Maxakalisaurus topai* Kellner *et al*., 2006; *Mendozasaurus neguyalap* Gonzalez-Riga, 2003; *Muyelensaurus pecheni* Calvo *et al*., 2007; *Narambuenatitan palomoi* Filippi *et al*., 2011; *Neuquensaurus australis* (Lydekker, 1893); *Opisthocoelicaudia skarzynskii* Borsuk-Białynicka, 1977; *Petrobrasaurus puestohernandezi* Filippi *et al*., 2011; *Overosaurus paradasorum* Coria *et al*., 2013; *Puertasaurus reuili* Novas *et al*., 2005; *Quetecsaurus rusconii* González-Riga and Ortiz David, 2014; *Rapetosaurus krausei* Curry-Rogers and Foster, 2001; *Rinconsaurus caudamirus* Calvo and González-Riga, 2003; *Rukwatitan bisepultus*, Gorsack *et al*., 2014; *Ruyangosaurus giganteus*; *Saltasaurus loricatus* Bonaparte and Powell, 1981; *Tapuiasaurus macedoi*; *Trigonosaurus pricei*; *Uberabatitan riberoi*; *Yongjinglong datangi* Li *et al*., 2014.

*Austroposeidon* is also compared with non-titanosaurian species, as follows: *Apatosaurus ajax* Marsh, 1877; *Apatosaurus excelsus* Marsh, 1879; *Camarasaurus supremus* Cope, 1877, *Diplodocus carnegii* Hatcher, 1901; *Giraffatitan brancai* (Janesch, 1914); *Mamenchisaurus youngi* Pi *et al*., 1996; *Qiaowanlong kangxii* You and Li, 2009.

### Heuristic tree search

The dataset (List A and Table A in S1 File) was analyzed using equally weighted parsimony in TNT [40] with a heuristic search of 1,000 replicates of Wagner trees followed by tree bisection-reconnection (TBR) branch swapping.

### Nomenclatural Acts

The electronic edition of this article follows the requirements of the amended International Code of Zoological Nomenclature. Also, the new names contained herein are available under the ICZN requirements from the electronic edition of this article. This published work and the nomenclatural acts it contains have been registered in ZooBank, the online registration system for the ICZN under the Life Science Identifiers code (LSIDs) specifications. The present work has the following LSID: urn:lsid:zoobank.org:pub:C9ACDD5F-BC33-4696-8ED0-36D3FA5B3AF8.

The electronic edition of this work was published in a journal with an ISSN, and has been archived and is available from the following digital repositories. All permits were obtained for the described study, which complied with all relevant regulations. See appropriate data about specimen numbers, locality, stratigraphy and repository in the “Results” section.

### CT Scan Imaging

The radiographic techniques discussed herein were performed using a General Eletrics LightSpeed 16 slice scanner at 120 kVp and 320mA. The analysis was performed in the Centro de Pesquisas da Petrobras (CENPES), at the Universidade Federal do Rio de Janeiro campus in Rio de Janeiro City. In most cases, the illustrations derived from the CT scans do not include raw data. Data were reconstructed in a free software 3D slicer, version 4.4.0 [41]. The 3D pdf (reconstruction pdf A and reconstruction pdf B in S2 File) was generated with MikTeX 2.9.5721 [42].

### Institutional abbreviations

**CENPES**—Centro de Pesquisas da Petrobras, Universidade Federal do Rio de Janeiro campus, Rio de Janeiro City.

**MCT**—Museu de Ciências da Terra, Rio de Janeiro, Brazil.

## Results

### Systematic Paleontology

Saurischia Seeley, 1887

Sauropodomorpha Huene, 1932

Sauropoda Marsh, 1878

Titanosauria Bonaparte and Coria, 1993

*Austroposeidon magnificus* gen. et sp. nov.

ZooBank Life Science Identifier (LSID) for the genus: urn:lsid:zoobank.org:act:8E496437-EA4E-4359-AA33-D792E6D70F95

***Austroposeidon*** new genus

**Type species:** *Austroposeidon magnificus* sp. nov., type by monotypy.

**Etymology**: “*Austro*”, meaning “Southern” in allusion to South America; and “*Poseidon*”, in reference to the Greek God responsible for earthquakes.

**Diagnosis:** The same as for the species

***Austroposeidon magnificus*** new species

ZooBank LSID for the species: urn:lsid:zoobank.org:act:BDD6403B-8AB8-4C7E-B7C2-EBBC400A825A

**Etimology**: The adjective “*magnificus*” (from the Latin), means “great, elevated, noble” in allusion to the large size of the specimen.

**Holotype**: MCT 1628-R, which is composed of two incomplete cervical vertebrae, one cervical rib, one dorsal vertebra, seven fragments of dorsal vertebrae and a fragment of a sacral vertebra.

**Diagnosis**: The new titanosaur is characterized by the following autapomorphies: 1) columnar-like centropostzygapophyseal laminae (cpol) in the last cervical vertebrae (Cv 13); 2) last cervical vertebra bearing a bifurcated posterior centrodiapophyseal lamina (pcdl); 3) first dorsal vertebra with the anterior and posterior centrodiapophyseal laminae (acdl/pcdl) curved ventrolaterally and with the diapophysis reaching the dorsal margin of the centrum; 4) the anteriormost portion of the spinoprezygapophyseal laminae (sprl) forked in the posterior dorsal vertebra.

The new species described here, can be further distinguished from other titanosaurs by the following combination of characters: presence of medial crest placed on the ventral surface of the last cervical centrum; presence of a suprapleurocoel lamina limiting the pleurocoel from the centrodiapophyseal fossae in the last cervical vertebrae; presence of developed centrodiapophyseal fossa in the posterior cervical vertebra; posterior cervical vertebrae with tall neural spines; presence of triangular centropostzygapophyseal fossae around the neural channel in the posterior cervical vertebra; robust spinoprezygapophyseal laminae in the anterior dorsal vertebrae; developed spinodiapophyseal laminae in the anterior dorsal vertebrae; strongly developed postzygaphophysis in the first dorsal vertebra; neural spine of the first dorsal vertebrae in vertical position and anteriorly located; prespinal lamina in the anterior dorsal vertebrae well developed until the base of the neural spine; diapophyses in the anterior dorsal vertebrae expanded anteroposteriorly and well inclined ventrolaterally; presence of well-developed pneumatizated camellae tissue in the cervical and dorsal vertebrae [43, 44]; absence of hyposphene-hypantrum articulation in the dorsal vertebra (*sensu* [45]); short and robust cervical ribs [46].

**Horizon and locality:** According to Campos and Castro [28], the material was found at the outskirts of the Presidente Prudente City, southwestern São Paulo State. According to the catalog of the Museu de Ciências da Terra (MCT—Museum of the Earth Sciences) the specimen was found at the Raposo Tavares road (BR-374), close to the Assis Chateaubriand Road (SP-425). The deposits of this region consist of sandstones and mudstones, and are referred to the Presidente Prudente Formation [29], which is considered Campanian-Maastrichtian in age [32]. One of us (FMS) tried to relocate the exact site from where this specimen was collected, but the area is nowadays urbanized.

### Description and Comparisons

#### Taphonomic remarks

All elements from the holotype and only known specimen of *Austroposeidon magnificus* have the shape altered to some degree due to taphonomy. The vertical axis tends to be twisted and compressed, and the material shows some taphonomic fractures. The cortical bone of several elements was partially lost, showing the internal camellae. The fragmentary nature of the material suggests that at least some breakages are the result of weathering indicating recent exposure, while others might have been caused during the collecting process. It is possible that more remains of this specimen were left at the outcrop.

Due to the anatomical features, size and collecting data, all vertebral elements are regarded to represent the same individual. The specimen is preserved in fine sandstone with cross lamination, indicating that it was deposited in a low energy flow regime, likely a crevasse splay of a floodplain.

#### Cervical vertebrae—general remarks

The preserved cervical vertebrae of *Austroposeidon magnificus* belong to the posterior region of the neck and were identified as most likely cervical 12 (Cv12) and cervical 13 (Cv13). An isolated cervical rib was also found, but it is not clear if it belongs to those elements. The cervical centra of Cv12 and Cv13 are incomplete and composed of the anteriormost and posteriormost part, respectively. They are markedly opisthocoelous, dorsoventrally depressed, and anteroposteriorly short. The dorsoventrally depressed centrum is a feature shared with *Ampelosaurus* [21], *Alamosaurus* [47, 48], *Futalognkosaurus* [3,4], *Isisaurus* [10], *Ligabuesaurus* [49], *Malawisaurus* [13], *Mendozasaurus* [50,51], *Puertasaurus* [52], and *Rinconsaurus* [53].

Medially to each spinoprezygapophyseal lamina, there is a depression at the base of that lamina. The neural arch of Cv12 is represented by only the left prezygapophysis and the basal portion of the neural spine. Cv13 shows the complete posterior portion of neural arch, with neural canal, both postzygapophyses and the part of the neural spine.

#### 12th Cervical vertebra

**Fig 2:**
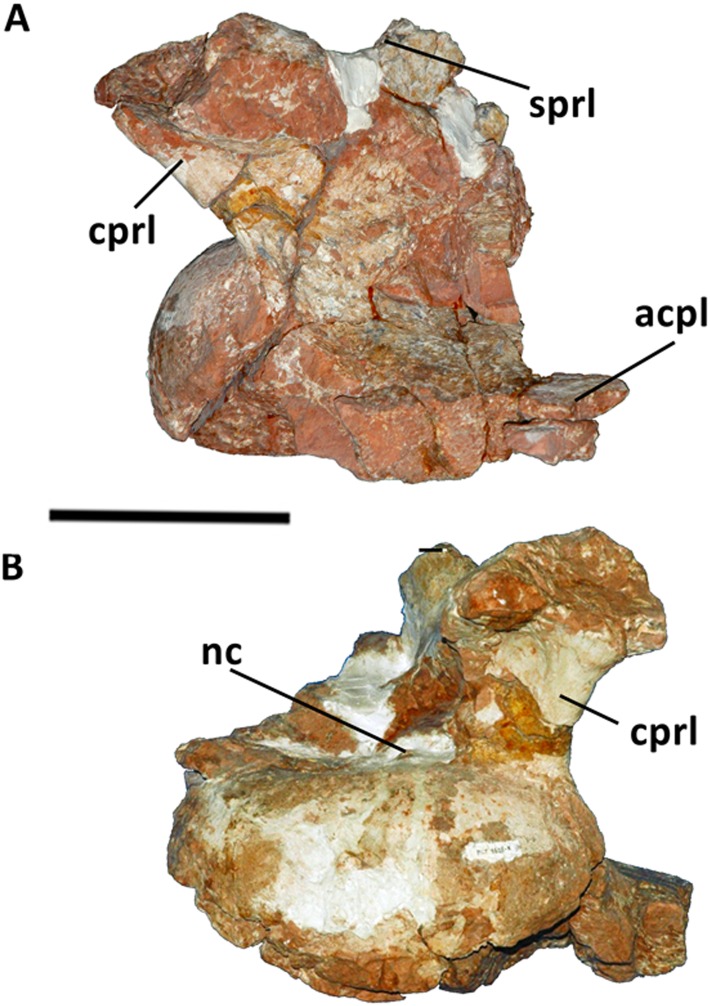
Cervical vertebra (Cv 12) of *Austroposeidon magnificus* gen. et nov. sp. (A) Left lateral and (B) anterior views. Abbreviations: acdl, anterior centrodiapophyseal lamina; acpl, anterior centroparapophyseal lamina; cprl, centroprezygapophyseal lamina; d, diapophysis; prz, prezygapophysis; prdl, prezygodiapophyseal lamina; prsl, prespinal lamina; s, neural spine; sprl, spinoprezygapophyseal lamina. Scale bar: 100mm.

The anterior articulation surface of the centrum of the 12th cervical vertebra is strongly convex. The ventral surface is poorly preserved on its anteriormost portion and there is no evidence of a ventral keel. The diapophysis is not preserved either. The anterior centroparapophyseal lamina is preserved only on the left side, is directed posteriorly and oriented parallel to the axis of the vertebral column (Fig 2A and 2B). The left prezygapophysis is well developed and inclined anterodorsally, slightly surpassing the anterior articulation surface of the centrum. On the right side, only the centroprezygapophyseal lamina, which is well developed, is preserved. There is no sign of an intraprezygapophyseal lamina. A portion of the prezygodiapophyseal lamina is preserved on the left side and, although not complete, it is clearly a robust structure. The spinoprezygapophyseal lamina is preserved on the left side and differs from *Futalognkosaurus dukei* [4] by being more robust. The anterior and the posterior centroparapophyseal laminae are laterally oriented, displaced anteriorly and well developed. The neural canal is deformed, probably by post-depositional stress.

#### 13th Cervical vertebra

**Fig 3:**
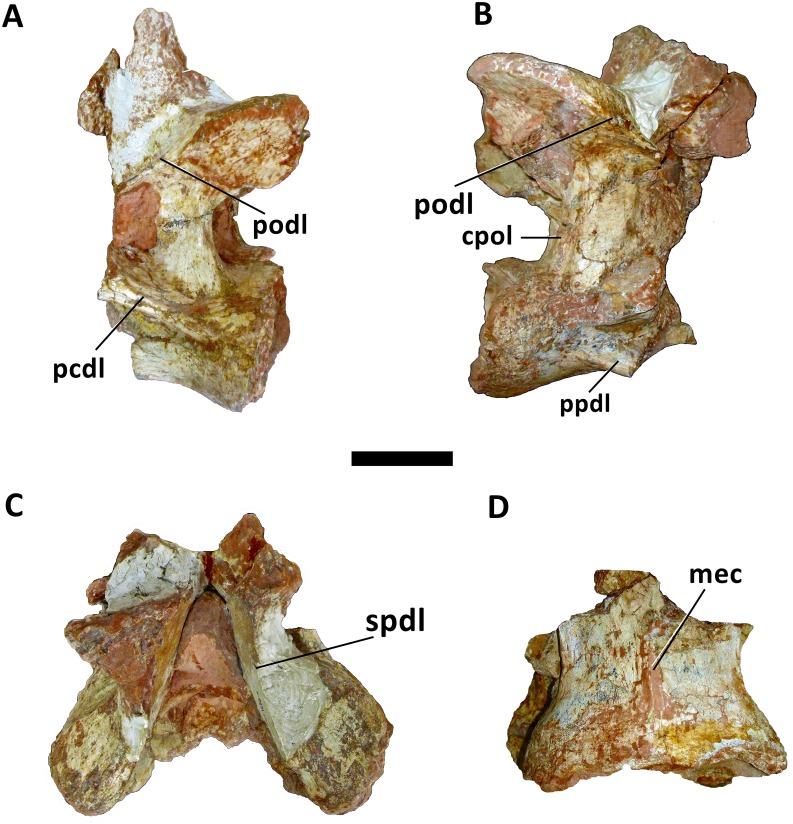
Cervical vertebra (Cv 13) of *Austroposeidon magnificus* gen. et sp. nov. (A) In left lateral, (B) right lateral, (C) dorsal and (D) ventral views. Abbreviations: cpol, centropostzygapophyseal lamina; mec, medial crest; pcdl, posterior centrodiapophyseal; podl, postzygodiapophyseal lamina; ppdl, paradiapophyseal lamina; spol, spinopostzygapophyseal lamina. Scale bar: 100mm.

The 13^th^ cervical has a maximum preserved height of 480 mm (Fig 3A and 3B). It is incomplete and lacks the anterior portion. The neural arch is two times higher than the centrum, similar to *Futalognkosaurus dukei* [4] and *Rapetosaurus krausei* [54]. Due to the inclination of the posterior centrodiapophyseal lamina (pcdl) and the postzygodiapophyseal lamina (podl) we assume that this bone is the last cervical vertebra.

Both postzygapophyses are wide and laterally expanded, being laterally oriented, with the articular surface flat and inclining laterodorsally (Fig 3A and 3B). They differ from the postzygapophyses of *Brasilotitan nemophagus* [7], *Trigonosaurus pricei* [55], MCT 1487-R [56], and *Uberabatitan riberoi* [57], for having the articular facets placed more laterally. In *Austroposeidon magnificus*, as in *Maxakalisaurus topai* [58], the processes of the postzygapophyses display large articulation facets. The neural spine of *Austroposeidon magnificus* is undivided (Fig 3C).

Although not complete, the preserved basal portion of the neural spine indicates that it should be taller, and therefore different from the condition reported in *Overosaurus paradasorum* [6], *Brasilotitan nemophagus* [7], *Malawisaurus dixeyi* [13], *“Aeolosaurus” maximus* [26], *Alamosaurus sanjuanensis* [47, 48], *Puertasaurus reuli* [52], *Rinconsaurus caudamirus* [53], *Trigonosaurus pricei* [55], MCT 1487-R [56], *Uberabatitan riberoi* [57], *Maxakalisaurus topai* [58], *Neuquensaurus australis* [59], *Petrobrasaurus puestohernandezi* [60], *Muyelensaurus pecheni* [61], *Narambuenatitan palomoi* [62], *Atsinganosaurus velauciensis* [63], and *Saltasaurus loricatus* [64, 65]. Similar to *Quetecsaurus rusconii*, [2] *Futalognkosaurus dukei* [3, 4], *Isisaurus colberti* [10], and *Ligabuesarus leanzai* [49], the neural spine of *Austroposeidon magnificus* is located at the posterior portion of the neural arch. The preserved portion of the neural spine has a lateral constriction near its base suggesting that the dorsal portion (not preserved) is expanded, similar to *Quetecsaurus* [2], *Futalognkosaurus* [3,4], *Ligabuesaurus* [49], *Mendozasaurus* [50, 51], *Puertasaurus* [52], and *Bonitasaura* [66, 67].

Ventrally, there is a constriction shortly after the posterior articulation surface which is positioned at the same level as the spinopostzygapophyseal laminae and at the level of a fossa delimited by the postzygodiapophyseal and the posterior centrodiapophyseal laminae. An incipient medial crest at the ventral surface (also described as ventral keel [6] or sagittal crest [33]), is observed shortly after the posterior articulation surface of the centrum (Fig 3D). This feature is also shared by the medio-posterior cervical of *Mendozasaurus neguyelap* [51], by the sole known cervical of *Gondwanatitan faustoi* [32], and the 12^th^ cervical vertebra of *Overosaurus paradasorum* [6]. In *Mendozasaurus neguyelap*, however, this medial crest is limited to the anterior region of the vertebra [50,51], while in *Austroposeidon magnificus* it reaches the posterior region of centrum (the anterior portion is not preserved). The new species also differs from *Gondwanatitan faustoi* by the absence of the two unnamed fossae delimited by this medial crest.

In lateral view, the posterior centrodiapophyseal and the posterior centroparapophyseal laminae are observed and are better preserved on the right side. The posterior centrodiapophyseal lamina is laterally expanded, well defined and positioned more posteriorly compared to *Quetecsaurus rusconii* [2], *Futalognkosaurus dukei* [4], *Overosaurus paradasorum* [6], MCT 1487-R [56], *Muyelensaurus pecheni* [61], and *Atsinganosaurus velauciensis* [63]. *Austroposeidon magnificus* shows a bifid posterior centrodiapophyseal lamina, a unique feature of the new species. This bifurcation starts at the posteriormost portion, from which each segment is inclined dorsally and diverge. *Austroposeidon magnificus* has the posterior centrodiapophyseal lamina strongly developed, similar to *“Aeolosaurus” maximus* [26]. The posterior centroparapophyseal laminae project ventrolaterally and differ from the ones of *Muyelensaurus pecheni* [61] by being more inclined.

**Fig 4:**
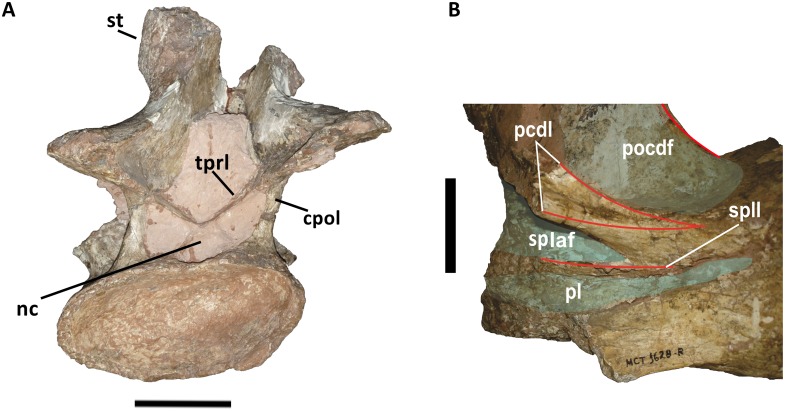
Detail of the cervical vertebra (Cv 13) of *Austroposeidon magnificus* gen. et sp. nov. (A) Lateral view, showing some of the new features of the species such as the bifurcated posterior centrodiapophyseal lamina (pcdl), the suprapleurocoel lamina (spll), and the suprapleurocoel cavity (splaf). (B) Posterior view. Abbreviations: cpol, centropostzygapophyseal lamina; nc, neural channel; pcdl, posterior centrodiapophyseal; pl, pleurocoel; poz, postzygapophysis; pocdf, postzygapophyseal centrodiapophyseal fossa; splaf, suprapleurocoel accessory fossa (new); spll, suprapleurocoel lamina (new); st, spine tuberosity; tpol, intrapostzygapophyseal lamina. Scale bar: 100mm.

*Austroposeidon magnificus* shows a lamina named here the suprapleurocoel lamina (spll; Fig 4), that is parallel to the main axis of the centrum. The suprapleurocoel lamina also delimitates two cavities: the pleurocoel and the suprapleurocoel accessory fossa (splaf), which is placed above the latter. The suprapleurocoel lamina of the new taxon differs from the internal septa present in various neosauropods (e.g. [50, 68]) by being connected to the posterior centrodiapophyseal lamina.

The centropostzygapophyseal laminae are tall, strongly developed and vertically oriented, and columnar-like, with proximal and distal expansions, resembling an iconic Greek column. The height of these laminae is followed by the development of the neural arch, a condition present in several neosauropods. However, this development is absent in Titanosauria, except for *Isisaurus* and *Ampelosaurus*. In the new taxon, the proximal and distal ends of the centropostzygapophyseal laminae are comparatively more expanded. Furthermore, the centropostzygapophyseal laminae of *Austroposeidon magnificus* reach almost the same height of the centrum, a unique feature of this taxon (Fig 3; Table 1). The posterior centrodiapophyseal laminae are well developed, a feature also observed in *Uberabatitan* (specimen 1108-UrB; [58]).

**Table 1:** Measurements of the major elements of *Austroposeidon magnificus* gen. et sp. nov. Dashed lines indicate portions which could not be measured since they are not preserved.

	Total height preserved	Preserved length	Preserved width	Condyle height	Condyle width	Cotyle height	Cotyle width
Cv 12	460mm	257mm	370mm	184mm	325mm	---	---
Cv 13	480mm	279mm	478mm	---	---	185mm	327mm
D1	462mm	510 mm	810 mm	187mm	329mm	---	---

The spinopostzygapophyseal laminae are short and thin, contacting the basal portion of the neural spine. They extend from the posteriormost portion close to the postzygapophyses, to the base of the neural spine, where they curve strongly upwards, getting more robust. These laminae are similar to the ones present in the last cervical vertebrae of *Quetecsaurus rusconii* [2] and *Malawisaurus dixeyi* [13], but differ from *Futalognkosaurus dukei* [4], *Mendozasaurus neguyelap* [51], and *Bonitasaura salgadoi* [66, 67] where they are less curved.

In dorsal view, this specimen presents a deep and narrow, spinopostzygophyseal fossa (Fig 3C). The posterior centrodiapophyseal and the postzygodiapophyseal laminae margin the postzygapophyseal centrodiapophyseal fossa, which is the dorsoventrally expanded. The postzygodiapophyseal laminae vary slightly in thickness. The posterior centroparapophyseal lamina is located at the most posterior half of the centrum, originating at the posterior articulation and becoming more developed at the most anterior preserved region.

The intrapostzygapophyseal lamina is "V" shaped, forming a 90° angle above the neural channel. The centropostzygapophyseal fossae present lateral to the neural channel are roughly triangular. The presence of the centropostzygapophyseal fossae in at least the last cervical and the anterior dorsal vertebrae is well documented in sauropods. However, regarding titanosaurians, these fossae have so far only been reported in dorsal vertebrae, and, contrary to the condition of the new taxon, are generally asymmetric [50, 62].

The presence of a "V" shaped intrapostzygapophyseal lamina and symmetrical centropostzygapophyseal fossae is observed in diplodocoids [69, 70], in the lithostrotians *Overosaurus paradosorum* [6] and *Rapetosaurus krausei* [54]; and in the macronarians *Camarasaurus supremus* and *Giraffatitan brancai* [39]. The macronarian *Qiaowanlong kangxii* shows same very prominent inclination of the intrapostzygapophyseal laminae however without the centropostzygapophyseal fossae [23].

#### Cervical Rib

**Fig 5:**
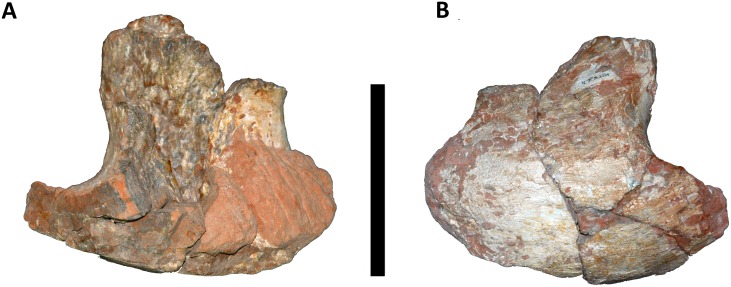
Cervical rib of *Austroposeidon magnificus* gen. et sp. nov. (A) Internal and (B) external views. Scale bar: 100mm.

**Fig 6:**
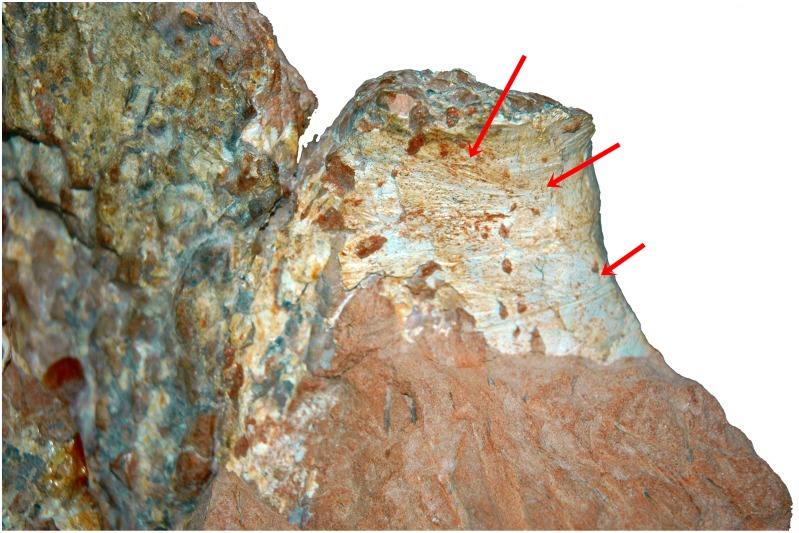
Detail of the cervical rib of *Austroposeidon magnificus* gen. et sp. nov. Red arrows show the scars for connective tissue. Scale bar: 50mm.

The sole cervical rib preserved in MCT 1628-R belongs to the left side and is fragmentary, comprising essentially the anterior region. It is a large and robust element, with a short and laterally compressed anterior process, and a convex lateral surface, contrasting with the concave medial surface (Fig 5A and 5B). A deep scar which probable was the attachment surface of connective tissue is observed on the right side (Fig 6). The head of the cervical rib is rounded similar to *Isisaurus colberti* [10] and more robust than in most other titanosaurs (e.g., [13, 55, 58]). The cervical rib is distinctive from *Overosaurus paradasorum* [6] and *Trigonosaurus pricei* [55] by having the anterior projection of the cervical rib less developed and differs from the cervical ribs of *Trigonosaurus pricei* [55], *Uberabatitan riberoi* [57], *Maxakalisaurus topai* [58], and *Petrobrasaurus puestohernandezi* [60] where the anterior and posterior processes are more elongated.

The cervical ribs of *Malawisaurus dixeyi* [13] and “*Aeolosaurus” maximus* [26] differ from the new taxon by be having the posterior portion narrower.

#### Dorsal Vertebrae

**Fig 7:**
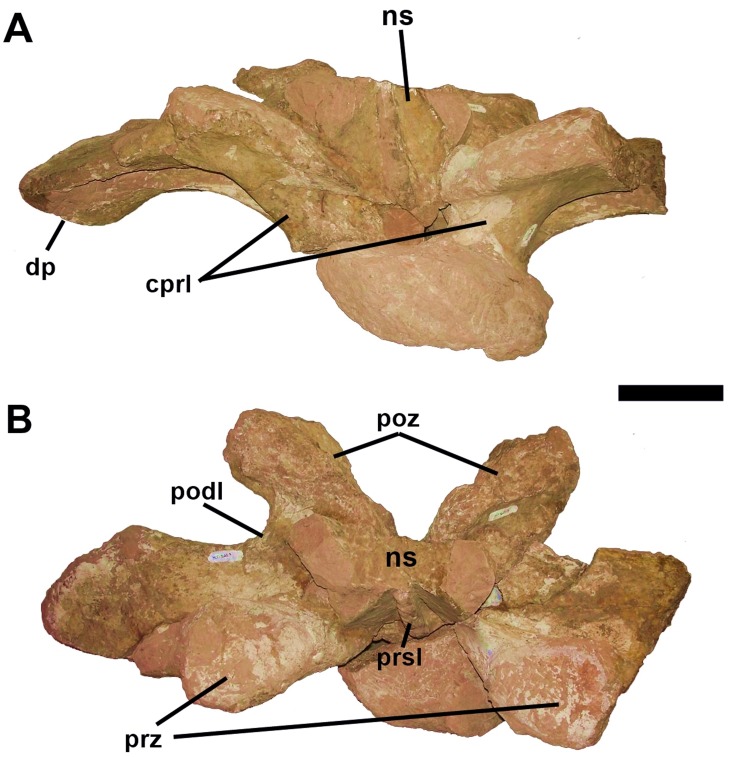
First dorsal vertebra (D1) of *Austroposeidon magnificus* gen. et sp. nov. (A) In anterior, (B) left anterolateral, (C) dorsal, (D) and posterior views. Abbreviations: cprl, centroprezygapophyseal lamina; ns, neural spine; prsl, prespinal lamina; prz, prezygapophysis; poz, postzygapophysis; spol, spinepostzygapophyseal lamina. Scale bar: 100mm.

**Fig 8:**
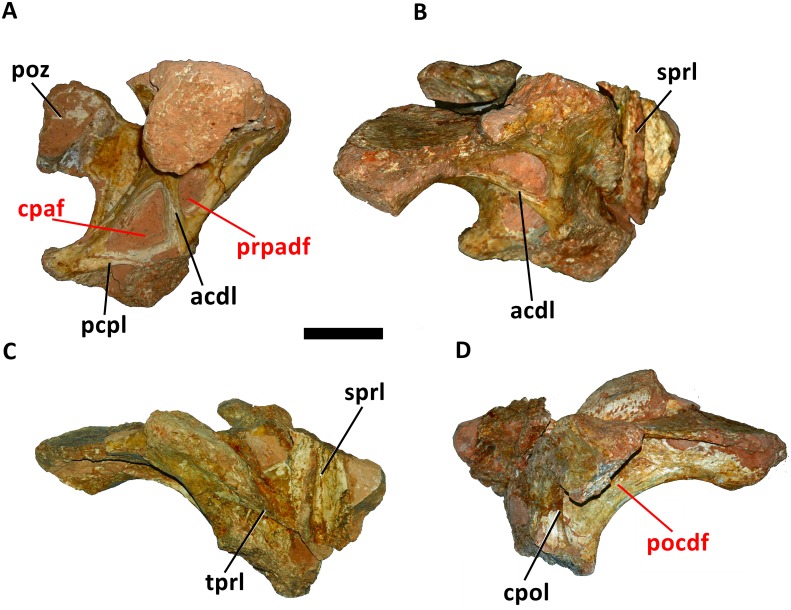
The dorsal vertebra of *Austroposeidon magnificus* gen. et sp. nov, major laminae and fossae. (A) In left anterolateral, (C) dorsal and (D) posterior views. The fossae are shown by red arrows. Abbreviations: acdl, anterior centrodiapophyseal lamina; cpaf, centroparapophyseal fossa; cpol, centropostzygapophyseal lamina; cprl, centroprezygapophyseal lamina; pcdl, posterior centrodiapophyseal; pcpl, posterior centroparapophyseal lamina; prsl, prespinal; poz, postzygapophysis; prcdf, prezygapophyseal centrodiapophyseal fossa; pocdf, postzygapophyseal centrodiapophyseal fossa; podl, postzygodiapophyseal lamina; tprl, intraprezygapophyseal lamina. Scale bar: 100mm.

All dorsal vertebrae are incomplete and their height cannot be established. The most complete is the first dorsal vertebra (D1), which is described here, has an almost complete neural arch and the base of the neural spine, well preserved prezygapophyses and postzygapophyses and the transverse processes (Figs 7A, 7B and 8). The neural arch of D1 is anteroposteriorly compressed, as in *Futalognkosaurus dukei* [4], *Isisaurus colberti* [10], *Yongjinglong datingi* [25], *Mendozasaurus neguyelap* [50,51], *Narambuenatitan palomoi* [63], *Argyrosaurus superbus* [71], and *Elaltitan lilloi* [72]. The preserved portion of the neural spine indicates that it is single differing from *Opisthocoelicaudia skarzynskii* [73], and vertically oriented (Fig 7A) similar to *Dreadnoughtus schrani* [8], *Argentinosaurus huinculensis* [46], *Mendozasaurus neguyelap* [50,51], and *Puertasaurus reuili* [52]. The neural spine is positioned more anteriorly on the neural arch in contrast to all other titanosaurs.

The prespinal lamina is robust, well defined, starting at the base of the neural spine and does not contact the intraprezygapophyseal lamina, differing from *Malawisaurus dixeyi* [13], but similar to the condition observed in many other titanosaurs, including *Mendozasaurus* [50,51]. Due to the incomplete nature of the dorsal vertebrae, it is not possible to establish if *Austroposeidon magnificus* shows as a postspinal lamina, which is a feature absent in titanosaurs (e.g., [10, 13, 52, 55, 56]).

The new taxon lacks a hyposphene-hypantrum articulation, differing from the condition reported in *Argentinosaurus huinculensis* [46], *Ligabuesaurus leanzai* [49], *Elaltitan lilloi* [72], *Epachthosaurus sciuttoi* [74], and *Andesaurus delgadoi* [75].

As in the cervical vertebrae, the zygapophyses display wide articulation facets disposed laterally. The articular facets of the prezygapophyses are oval and well separated from each other, similar to the second dorsal of *Puertasaurus reuili* [52] and the 4^th^ dorsal of *Neuquensaurus australis* [60]. In the new taxon, as well as in *Argentinosaurus huinculensis* [46], *Mendozasaurus neguyelap* [50,51], and *Bonitasaura salgadoi* [67], the prezygapophyses are inclined ventrally. The postzygapophysis is wider than high and displays an elongated and flattened articulation facet which is smaller than in the prezygapophysis (Fig 7B). The articular facet is directed ventromedially, as in *Mendozasaurus neguyelap* [50, 51] and *Muyelensaurus pecheni* [61].

The transverse processes are displaced ventrolaterally with their distal ends strongly inclined ventrally, resembling *Mendozasaurus neguyelap* [50,51] (Fig 7A and 7B). The transverse process is expanded anteroposteriorly like in *Futalognkosaurus dukei* [4] *Ligabuesaurus leanzai* [49] and *Mendozasaurus neguyelap* [50,51]. The diapophysis is also expanded anteroposteriorly, more than in other titanosaurs, and are directed ventrolaterally. The centroprezygapophyseal and the centropostzygapophyseal laminae are vertical and very strong, similar to the condition of those laminae in the posterior dorsals of *Futalognkosaurus dukei* [4], *Malawisaurus dixeyi* [13], *Ligabuesaurus leanzai* [49], *Puertasaurus reuili* [52], and *Muyelensaurus pecheni* [61]. The transverse process is reinforced by the curved centrodiapophyseal lamina, differing from all other titanosaurs (Fig 7A and 7B).

The intraprezygapophyseal lamina is poorly developed. It is directed downward, reaching the prezygapophysis laterally, joining the basal portion of the neural spine. The spinoprezygapophyseal lamina also connects the basal portion of the neural spine but does not reach the articular facet of the prezygapophysis. The postzygodiapophyseal lamina is slender and short, similar to the condition observed in the 9^th^ and 10^th^ dorsal vertebrae of *Trigonosaurus pricei* [55]. The spinopostzygapophyseal lamina is short and robust, comparable to condition found in the posterior cervical vertebra. The centropostzygapophyseal lamina is slender and positioned just below the postzygapophysis. As proposed by Wilson and colleagues [39], the infradiapophyseal cavity is identified here as the infradiapophyseal fossa, and differs from the infradiapophyseal depression of *Muyelensaurus pecheni* [61] and *Rinconsaurus caudamirus* [52], where it is slightly shallower than in the new taxon.

The spinodiapophyseal fossa is dorsomedially expanded, but less than in *Futalognkosaurus dukei* [4] and *Mendozasaurus neguyalap* [50,51]. The prezygapophyseal centrodiapophyseal fossa (prcdf) is deep with an elongated shape, differing from *“Aeolosaurus” maximus* [26], *Gondwanatitan faustoi* [32], *Trigonosaurus pricei* [55], MCT 1487-R [56], and *Uberabatitan riberoi* [57]. The elongated shape of the prezygapophyseal centrodiapophyseal fossa (prcdf) is similar to D1 of *Maxakalisaurus topai* [58]. The new taxon, however, lacks a second fossa placed immediately below the diapophysis observed in the latter (KLNB personal observation). The prezygapophyseal centrodiapophyseal fossa (prcdf) is more developed dorsoventrally than the parapophyseal centrodiapophyseal fossa (pacdf; Fig 8A and 8B). The latter (pacdf) is subtriangular, deep and more developed dorsoventrally than anteroposteriorly. The postzygapophyseal centrodiapophyseal fossa (pocdf) is the most elongated of the previous ones (Fig 8D), similar as in *Uberabatitan riberoi* [57].

Besides the first dorsal vertebra (D1) fragments of neural arches of other dorsal vertebrae of *Austroposeidon magnificus* were identified. All show massive spongy bone structures (*camellae*).

Among the fragments there is a right transverse process of likely the 3^rd^ or 4^th^ anterior dorsal vertebra. It is very similar to the distal end of the transverse process of D1. The incomplete centrodiapophyseal lamina and the centrodiapophyseal fossa (filled with matrix) can be identified on the ventral side of this element. Based on the preserved portion, this fossa is reduced in this vertebra. No prezygapophyseal centrodiapophyseal and the centroparapophyseal fossae were observed and are likely absent.

**Fig 9:**
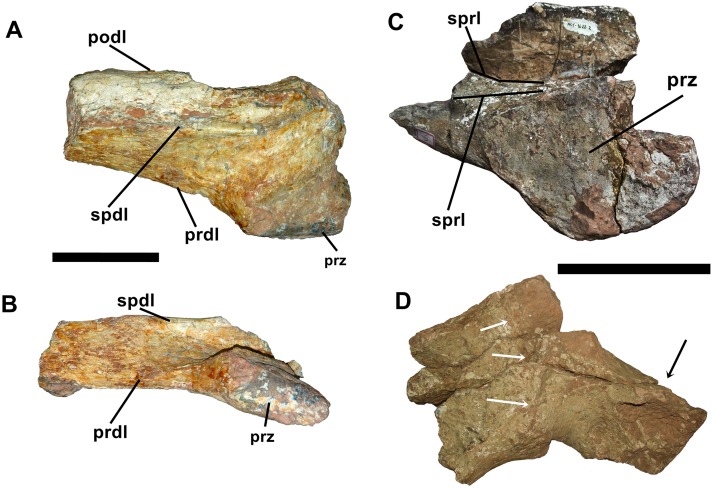
Additonal elements of dorsal and sacral vertebrae of *Austroposeidon magnificus* gen. et sp. nov. (A) Left transverse process of a posterior dorsal vertebra, in anterior, (B) posterior views. (C) Left prezygapophysis from the posterior dorsal vertebra, with dark lines showing the bifurcated spinoprezygapophiseal lamina. (D) Sacral element, with white arrows indicating the suture between the sacral transverse process and the sacral rib, and the black arrow the sacral rib. Abbreviations: prdl, prezygodiapophyseal lamina; prz, prezygapophysis; podl, postzygodiapophyseal lamina; prdl, prezygodiapophyseal lamina; spdl, spinodiapophyseal lamina; sprl, spinoprezygapophyseal lamina. Scale bar: 100mm.

A second incomplete element is interpreted as the left transverse process likely from the 5^th^ or the 6^th^ dorsal vertebra. The prezygapophysis is wide and oval-shaped. The centroprezygapophyseal lamina is inclined and located closer to the transverse process compared to the other preserved elements. The posterior centrodiapophyseal lamina is less developed than in the first dorsal (D1, Fig 9A). The spinoprezygapophyseal lamina is situated more distally compared to D1. The spinodiapophyseal lamina (spdl) is well developed and vertically oriented, similar to *“Aeolosaurus” maximus* [26] and *Maxakalisaurus topai* [58] (Fig 9B). It (spdl) forms, together with the spinoprediapophyseal lamina, the spinodiapophyseal fossa (sdf). The prezygapophyseal centrodiapophyseal fossa is reduced, shallow and has an elliptical shape.

An isolated left prezygapophysis likely belonging to the 7^th^ or the 8^th^ posterior dorsal vertebra was also identified. The spinoprezygapophyseal lamina is positioned distally relative to the prezygapophysis and together with the spinodiapophyseal lamina, forms the spinodiapophyseal fossa (sdf). This is the shortest spinodiapophyseal fossa in *Austroposeidon magnificus* and is more anteroposteriorly elongated than all other fossae described for this taxon. This fragment is notable for the bifurcation of the spinoprezygapophyseal lamina on the basal most portion (Fig 9C).

The last identifiable element of the dorsal series is the left prezygapophysis from likely the 9^th^ posterior dorsal vertebra. The prezygapophysis is small and has a rectangular shape. The centroprezygapophyseal lamina is placed laterally and more posteriorly than in other preserved elements of this taxon. The spinodiapophyseal fossa is shallow. It bears two laterally placed fossae separated by a thin bony lamina and might be the prezygapophyseal centrodiapophyseal and parapophyseal centrodiapophyseal fossae (Fig 9C).

#### Sacral vertebra

Only a fragmentary element of the sacral series could be identified. The material consists of the transverse process and the sacral rib, that is separated by a suture showing that they are not completely fused (Fig 9D). The sacral vertebra possesses a camellate internal structure. The preserved portion of the sacral rib is almost triangular in shape and strongly curved to upwards.

## Discussion

Despite incomplete, the specimen MCT 1628-R represents a new sauropod dinosaur from Brazil, named here *Austroposeidon magnificus* gen. et sp. nov. It can be classified within Titanosauria based on the lack of hyposphene-hypanthrum, single neural spine of the cervical and dorsal vertebrae and the camellate internal structure (see below). It can be separated from all other titanosaurs by several autapomorphies, which include the columnar-like centropostzygapophyseal laminae in the last cervical vertebrae and the presence of a bifurcated posterior centrodiapophyseal lamina (see diagnosis). The new species also comprises the largest dinosaur known from Brazil so far, with an estimated length from head-to-tail of around 25 meters. Although detailed ontogenetic stages have been recognized for titanosaurs (as recently attempted for other fossil reptiles [76], there are no signs of immaturity in the present specimen and we here regard MCT 1628-R as representing an adult individual.

*Austroposeidon magnificus* was collected in deposits referred to the Presidente Prudente Formation (e.g., [29]). This unit has also revealed the presence of two other titanosaurs: *Brasilotitan nemophagus* [7] and *Gondwanatitan faustoi* [32], both small-sized and considered derived members of this group of sauropods [3, 5, 7, 32]. *Austroposeidon* differs from those taxa mainly by the morphology of the cervical vertebrae, which are proportionally shorter, bear taller neural spines and robust centroprezygapophyseal and centropostzygapophyseal laminae. The prezygapophyses and postzygapophyses of the preserved elements of the new species are also wider, laterally expanded, and more inclined laterodorsally.

In order to establish the phylogenetic position of *Austroposeidon magnificus*, we have performed a cladistic analysis using mainly the dataset published by Gonzalez Riga and Ortiz David [2]. We have included five new characters and 15 additional taxa (plus *Austroposeidon magnificus*). One character was modified due to repetition and one excluded for being non-informative (List A and Table A in S1 File). This new analysis has the largest number of titanosaur species used in a phylogenetic analysis so far.

**Fig 10:**
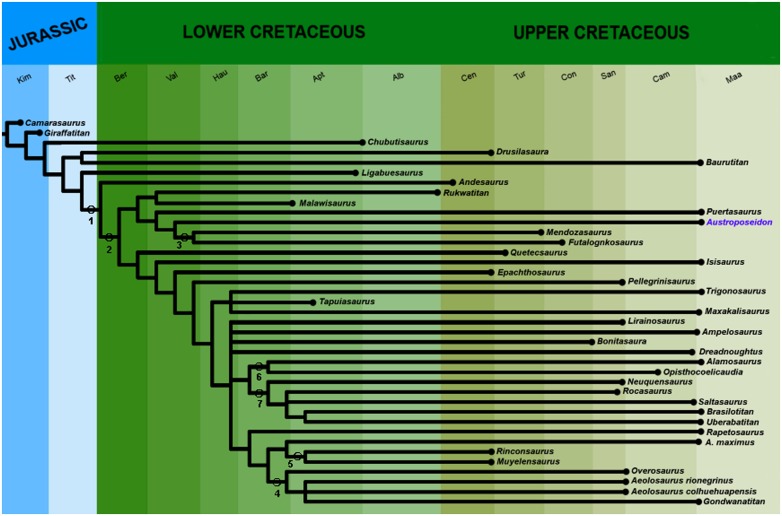
Strict consensus cladogram, showing the relationships of *Austroposeidon magnificus* gen. et sp. nov. The analysis generated two most parsimonious trees, with 233 steps, consistency index (C.I.) of 0.468, and retention index (R.I.) of 0.636. Nodes are as follows: 1) Titanosauria, 2) Lithostrotia, 3) Lognkosauria, 4) Aeolosaurini, 5) Rincosauria, 6) Opisthocoelicaudinae, and 7) Saltasaurinae.

The analysis was performed using TNT, version 1.1 [40], and multistate characters were considered unordered. The analyses generated two most parsimonious trees, with 233 steps, with consistency index (C.I.) as 0.468, and retention indexes (R.I.) as 0.636. The analyses of strict consensus are shown in Fig 10.

The new phylogenetic analysis recovered *Austroposeidon magnificus* as the sister-group of Lognkosauria (*Mendozasaurus* + *Futalognkosaurus*). This relationship is supported by one synapomorphy: presence of a ventral keel on the posterior cervical centra separating two concavities or fossae (character 30.1).

The present analysis also shows that *Puertasaurus* is closely related to the clade formed by *Austroposeidon* + Lognkosauria, supported here by two characters: the lateral laminae on the posterior cervical neural spines surpassing the width of the centra (character 32.1) and the posterior cervical centra proportions being less than 1,5 (character 34.2). Although the lateral lamina is less developed in *Puertasaurus* and *Austroposeidon* compared to the Lognkosauria, its presence has been associated with the large lateral development of the neck [4, 50, 51]. In all those taxa the shortly anteroposterior length of the vertebrae, especially the posterior cervicals and the anterior dorsals, contrast with the dorso-ventral elongation of the spinodiapophyseal fossae (sdf).

Traditionally, five different clades are recognized within Lithostrotia (e.g., [4, 7, 77]): Lognkosauria, Rinconsauria, Opisthocoelicaudinae, Aeolosaurini, and Saltasaurinae (Fig 10). All those clades are recovered in phylogenetic analysis presented here, but the relationships among them differ from previous studies. The clade Lognkosauria (plus *Puertasaurus* and *Austroposeidon*, both included for the first time in a phylogenetic analysis), for example, was recovered closer related to *Malawisaurus* + *Rukwatitan* than the analysis presented by Gorscak [14]. "*Aeolosaurus*" *maximus* was not recovered with the other species of *Aeolosaurus* and might potentially belong to a different genus. According to the previous studies, *Tapuiasaurus* was regarded as closely related to *Rapetosaurus* [5, 14], a relationship not recovered here. *Tapuiasaurus* turned out to in a trichotomy with *Trigonosaurus* and *Maxakalisaurus*. The clade *Uberabatitan* + *Brasilotitan* (also included for the first time in a phylogenetic analysis) was recovered as sister-taxa in the Saltasaurinae that includes *Rocasaurus* and *Saltasaurus*. The present study shows that there is much more work to be done in order to provide a more consistent proposal of the in-group relationships of titanosaurs, that might influence biogeographic studies as has been the case for other dinosaurs (e.g., [78]).

### CT Scan Analysis

The absence of 3D detailed internal investigation in many titanosaurs, like sauropods, hinders a detail correlation between internal and external pneumatic structures, especially in respect to the ontogenetic stages [79]. Recently, a few titanosaurs have been analyzed with CT scan [7, 8, 14, 80], but none have internal 3D models.

**Fig 11:**
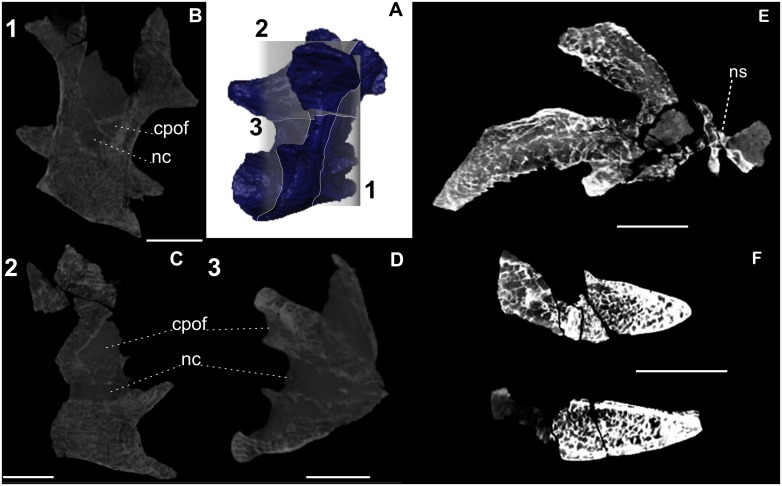
Pneumatic structures of *Austroposeidon magnificus* gen. et sp. nov. (A) The anatomical planes analyzed in this study: 1—Coronal, 2- Median, 3 –Transversal. (B) Coronal plane showing the extensive centro-postzygapophyseal fossae at the middle portion of the vertebra. (C) and (D) show the the centro-postzygapophyseal fossae and the neural canal, in median and transverse planes, respectively. (E) Dorsal vertebra (D1) in transverse plane. (F) Sacral vertebra in transverse plane. Note the internal pneumatic constructions showing a connection between smaller camellate structures, as evidenced by the white dashed lines. See text for discussion. Abbreviations: cpof, centropostzygapophyseal fossae; nc, neural channel, ns, neural spine. Scale bar: 100mm.

Two cervical (Cv 12 and Cv13), one dorsal (D1), and one sacral (Sc) vertebrae of *Austroposeidon magnificus* were CT-scanned in order to investigate internal structures. The Cv12, however did not provide good tomographic images due lack of contrast between matrix and bone. However, four features, never before reported in titanosaurs were observed: 1) in coronal axes the external posterior triangular centropostzygapophyseal fossae of the Cv13 is developed internally and persists to the third half of the vertebra (Fig 11); 2) trends of camellate rings are oriented anteroposteriorly; 3) rings of concentric camellate structures are limited by higher density tissue; 4) the intercalation of external high density tissue and internal pneumatic tissue are three times repeated in the fragmented vertebral lamina.

**Fig 12:**
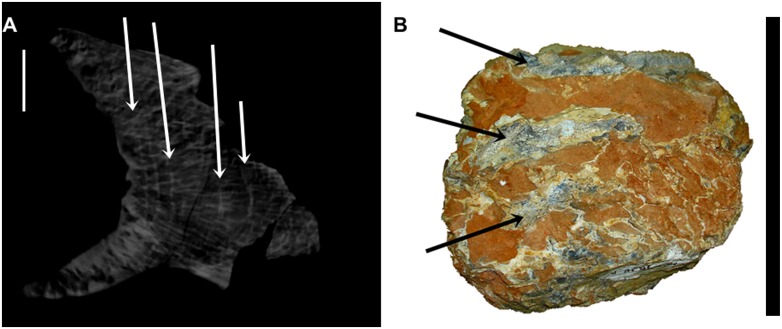
The camellate rings revealed by tomographic CT scans in cervical vertebrae. (A) The camellate rings Cv13 evidenced by the white arrows. Those structures are interpreted as growth structures. (B) The intercalation of external high density tissue (black arrows) and internal pneumatic tissue in an isolated fragment of a cervical vertebra. Scale bar: 50mm.

The camellate rings are well marked and are here interpreted as intercalated growth structures, which seem to be concentric in respect to the centrum of the vertebra (Fig 12A). Such intercalation of external high dense and internal pneumatic camellate tissues was also observed in a broken isolated fragment of a cervical vertebrae of this new species which was not submitted to CT scan analysis (Fig 12B).

**Fig 13:**
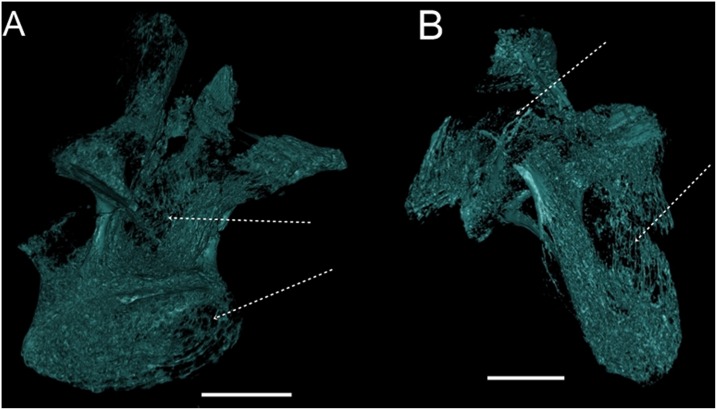
The tentatively 3D reconstruction of the internal pneumatic structures in *Austroposeidon magnificus* gen. et sp. nov. (A) The last cervical vertebra (Cv13) in posterolateral view. (B) First dorsal vertebra in left posterolateral view. The internal pneumatic connections suggest a possible interconnection of all the internal pneumatic structures throughout the entire vertebral body (light green). Scale bar: 100mm.

The tentatively 3D reconstruction of the internal pneumatic openings shows a connection between the smaller camellate structures, suggesting a possible interconnection of all internal pneumatic structures throughout the entire vertebral body (Fig 13, (reconstruction pdf A and reconstruction pdf B in S2 File). These mentioned features suggest a large diversity of pneumatic stages, which need to be investigated together with the internal osteological anatomy of fossil vertebrates.

## Conclusions

The description of this new species, *Austroposeidon magnificus*, increases our knowledge of Brazilian titanosaurs, particularly the giant ones, which have not been reported previously in this country. Despite the fragmentary condition of the new species, a phylogenetic analysis shows that *Austroposeidon magnificus* is the sister group of Lognkosauria, a clade that comprises other giant titanosaurs. CT scan analysis reveals some new information about internal anatomic features of large titanosaurs, including potential growth patterns. Some of those internal structures are here observed for the first time and reinforce the importance of the CT scan studies in those giant dinosaurs.

## Supporting Information

S1 File List A, Character List. Table A, Character matrix.
(DOC)

S2 File Reconstruction pdf A, The 3D reconstruction of Cv13. Reconstruction pdf B, The 3D reconstruction of D1.
(RAR)
