# Supplementary material for: A New Giant Titanosauria (Dinosauria: Sauropoda) from the Late Cretaceous Bauru Group, Brazil
Source: PLoS One. 2016 Oct 5;11(10):e0163373. doi: 10.1371/journal.pone.0163373 (PMC5051738; doi:10.1371/journal.pone.0163373)
Supplement: S1 File — List A, Character List. Table A, Character matrix. (DOC) [file pone.0163373.s001.doc]

**List A. Character List.**

This character list follows mainly Gonzalez-Riga and Ortiz David (2014). Character 24 of this list was excluded for being uninformative. Character 26 and 27 were united. Five new characters were included and some modified, as presented below.

1. Short, deep snout: present (0); absent (1) (Curry Rogers, 2005 - Character 1).
2. Frontal contribution to supratemporal fossa: present (0); absent (1) (Wilson, 2002 -Character 18).
3. Frontal, dorsal texture: smooth (0); rugose (1) (Gallina and Apesteguía, 2011- Character 3).
4. Postorbital, ventral process shape: transversely narrow (0); broader transversely than anteroposteriorly (1) (Wilson, 2002- Character 16).
5. Postorbital, posterior process: present (0); absent (1) (Wilson, 2002- Character 17).
6. Postorbital, posterior margin articulating with the squamosal: with tapering posterior process (0); with a deep posterior process (1) (Zaher et al., 2011- Character 245).
7. Parietal occipital process, dorsoventral height: short, less than the diameter of the foramen magnum (0); deep, nearly twice the diameter of the foramen magnum (1) (Wilson, 2002 - Character 21).
8. Parietal, elongate lateral process: absent (0); present (1) (Curry Rogers, 2005 - Character 37).
9. Parietal, cranial inclination with wide posterodorsal exposure of crest: absent (0); present (1) (Salgado and Calvo, 1997)
10. Parietal, contribution to post-temporal fenestra: present (0); absent (1) (Wilson, 2002 - Character 22).
11. Parietal, distance separating supratemporal fenestrae: less than (0); or twice (1) the long axis of the supratemporal fenestra (Wilson, 2002 - Character 24).
12. Ascending process of premaxilla: directed dorsally (0); directed posterodorsally (1) (Gauthier, 1986).
13. External nares, configuration of lateral margin: lacrimal excluded, maxilla-nasal contact (0); lacrimal participates, separates maxilla and nasal (1) (Gallina and Apesteguía, 2011 - Character 10).
14. Preantorbital fenestra: absent (0); present (1) (Wilson and Sereno, 1998 - Character 74).
15. Supraoccipital, height: twice (0); subequal to or less than (1) height of the foramen magnum (Wilson, 2002 - Character 43).
16. Paraoccipital process, ventral nonarticular process: absent (0); present (1) (Wilson, 2002 - Character 44).
17. Longitudinal groove on the supraoccipital: absent (0); present (1) (Gonzalez Riga and Ortiz David, 2014 - Character 17).
18. Angle between basipterygoid processes: is approximately 45º (0); is approximately 20º (1) (Salgado & Calvo, 1995).
19. Basal tubera, anteroposterior depth: approximately half the dorsoventral height (0); sheetlike, 20% the dorsoventral height (1) (Wilson, 2002 - Character 48).
20. Mandible shape: U shape (0); L shape (1) (Gallina and Apesteguía, 2011 - Character 17).
21. Tooth shape: spoon-like (0); compressed cone chisel-like (1); pencil chisel-like (2) (Calvo and González Riga, 2003 - Character 1).
22. Tooth crowns, cross-sectional shape at mid-crown: D-shaped (0); subcylindrical with smooth crest (1); cylindrical (2) (Calvo *et al*., 2007 - Character 10).
23. Wear facets of teeth sharply inclined: absent (0); present (1) (Calvo *et al*., 2007a - Character 9).
24. Cervical vertebrae, number: 12 (0); 13 (1); 14 or more (2) (Calvo *et al*., 2007a -Character 11).
25. Cervical neural spines height**: tall (0); low (1)** (modified from Calvo et al., 2007a - Character 16).
26. **Cervical neural spines, distal end with tubercle: absent (0); present, poor developed (1); present, well developed (2) (New).**
27. Relative length of cervical prezygapophyses: long, with articular facets anterior to diapophyses (0); short, with articular facets positioned near level of diapophyses (1) (Salgado *et al*., 1997 - Character 37).
28. Anterior cervical neural spines: transversely broad, laterally expanded (0); anteroposteriorly expanded and compressed transversely (1) (Curry-Rogers, 2005 - Character 124).
29. Anterior cervical centra, heigh width ratio: less than 1 (0); approximately 1.25 (1) (Wilson, 2002 - Character 84).
30. **Posterior cervical centra, ventral surface,** **midventral thin keel or a ridge: absent, with only concave surface (0); present, bearing two concavities or two fossae (1)** (modification from Curry-Rogers, 2005 - Character 118 by this research).
31. **Posterior cervical vertebrae, neural spines: transversely broad, laterally expanded (0); anteroposteriorly expanded and compressed transversely (1) (New).**
32. Lateral laminae on posterior cervical neural spines: **absent (0); present, equal or reaching the wide of the centra (1); present, surpassing the wide of the centra** (modified from Gonzalez-Riga and Ortiz David, 2014 - Charater 26).
33. Spinodiapophyseal fossa in cervical vertebrae: absent (0); shallow (1); deep and dorsally extended (2) (González Riga, 2005 - Character 9).
34. Posterior cervical centra, proportions: ratio anteroposterior length/ height of posterior face: >3 (0); 2,5 to 1,5 (1); less than 1,5 (2) (Calvo *et al*., 2007 - Character 20)
35. Dorsal vertebrae, number: 12 or more (0); 11(1), 10 or fewer (2) (Wilson and Sereno, 1998 - Character 70).
36. Prespinal lamina in dorsal vertebrae: absent (0); present only as an incipient prespinal lamina (1); present in the distal end of neural spine (2); well developed up to the base of the neural spine (3) (Bonaparte *et al.,* 2006 - Character 34).
37. Hyposphene-hypantrum articulation in dorsal vertebrae: present (0); absent (1) (Salgado *et al*., 1997 - Character 25).
38. Pleurocoels in dorsal vertebrae, shape: circular or elliptical (0); caudally acuminate (1) (Calvo *et al*., 2007 - Character 29).
39. Dorsal transverse processes: directed strongly dorsolaterally (0); directed laterally or slightly upward (1) (Curry Rogers, 2005 - Character 154).
40. Anterior dorsal neural spines: straight, dorsally directed (0); posteriorly inclined (1) (Curry-Rogers, 2005 - Character 151).
41. Anterior dorsal neural spines, shape: bifid (0); single (1) (McIntosh 1990).
42. Anterior dorsal vertebrae, infrapostzygapophyseal fossa: absent (0); present not divided (1); present, divided in two subtriangular fossae (2). (Gallina & Apesteguía, 2011 - Character 33).
43. Anterior dorsal neural spines inclined posteriorly more than 20º from vertical: absent (0); present (1) (González Riga, 2003 - Character 4).
44. Posterior dorsal neural spines, dorsal development: more (0); or less (1) than 20% the total height of the vertebra (Calvo *et al*., 2007a - Character 24).
45. Centroparapophyseal lamina in posterior dorsal vertebrae: absent (0); present (1) (Bonaparte *et al*., 2006 - Character 35).
46. Ventrally widened or slightly forked centrodiapophyseal laminae in posterior dorsal vertebrae: absent (0); present (1) (Calvo *et al.*, 2007 - Character 27).
47. Camellate or somphospondylous types of internal structures of presacral vertebrae: absent (0); present (1) (Bonaparte *et al*., 2006 - Character 40).
48. Sacral vertebrae, number: five (0); six of more (1) (McIntosh 1990).
49. First caudal vertebrae, type: platycoelous (0); procoelous (1); opisthocoelous (2); biconvex (3) (Calvo & Gonzalez Riga, 2003 - Character 20).
50. Wide and deep interzigapophyseal cavity in proximal caudal vertebrae: absent (0); present (1) (Calvo *et al.,* 2007a, 2007b - Character 33).
51. Anterior caudal centra, pneumatic fossae: absent (0); present (1) (Wilson, 2002 - Character 119).
52. Caudal transverse processes: persist through caudal 20 or more posteriorly (0); disappear by caudal 15 (1); disappear by caudal 10 (2) (Wilson, 2002 - Character 115).
53. Anterior and middle caudal centra, proportions: as high as wide (0); depressed, wider than high (1) (Calvo & Gonzalez Riga, 2003 - Character 22).
54. Mid caudal centra with the anterior face strongly inclined anteriorly: absent (0); present (1) (Franco-Rosas *et al*., 2004).
55. Articular face shape on middle caudal centra: non-procoelous (0); procoelous- distoplatyan (1); slightly procoelous (2); strongly procoelous with prominent condyles (3) (Gonzalez Riga *et al*., 2009 - Character 53).
56. Articular face shape on posterior caudal centra: non-procoelous (0); slightly procoelous with reduced condyles (1); strongly procoelous with prominent condyles (2) (Gonzalez Riga *et al*., 2009 - Character 54).
57. Anterodorsal border of neural spine in middle caudal vertebrae located posteriorly with respect to anterior border of the postzygapophyses: absent (0); present (1) (Calvo *et al*., 2007a - Character 40).
58. Anteriorly directed anterior caudal neural spine: absent (0); present (1) (Calvo *et al*., 2007 - Character 41).
59. Shape of the section of neural spines in most anterior caudal vertebrae in dorsal view: axially elongated (0); transversely elongated (1); quadrangular (2) (Calvo *et al*., 2007b - Character 42).
60. Neural arch in anterior caudal vertebrae: placed in the middle of the centrum (0); anteriorly (1); on the anterior border (2) (Calvo *et al*., 2007 - Character 39).
61. Neural spine in the middle caudal vertebrae, shape: short anteroposteriorly (0); laminated, anteroposteriorly elongated and posteriorly projected reaching the posterior border of the centrum (1); laminated, anteroposteriorly elongated and located over the middle of the centrum (2) (González Riga, 2003 - Character 21).
62. Length proportions of prezygapophyses with respect to the centrum length in middle caudal vertebrae: shorter than 50% (0); between 40 to 50% (1); longer than 50% (2) (Calvo *et al*., 2007 - Character 44).
63. Ventral depression divided by a longitudinal septum in anterior and middle caudal vertebrae: absent (0); present (1) (Salgado and Azpilicueta 2000).
64. Postzygapophyseal process in middle caudal vertebra: absent (0); present (1) (Calvo *et al*., 2007a, 2007b - Character 46).
65. Well-developed interprezigapophyseal lamina in middle caudal vertebrae: absent (0); present (1) (Calvo *et al*., 2007a, 2007b - Character 47).
66. Scapular glenoid orientation: relatively flat (0); strongly beveled medially (1) (González Riga, 2003 - Character 25).
67. Humerus, breadth of proximal end: less (0); equals or more than 50% (1) the humeral length (González Riga *et al*., 2009 - Character 75).
68. Humerus, type of proximal border: strongly curved (0); straight or slightly curved (1); sigmoidal (2) (González Riga, 2003 - Character 27).
69. Ulnar olecranon process, development: prominent, projecting above proximal articulation (0); rudimentary, levels with proximal articulation (1) (Wilson and Sereno, 1998 - Character 4).
70. Sternal plates, shape: suboval (0); semilunar (1) (Salgado *et al*., 1997 - Character 26).
71. Semilular sternal plate with straight posterior border: absent (0); present (1) (González Riga, 2003 - Character 29).
72. Coracoid, shape: suboval (0); quadrangular (1) (Salgado *et al.,* 1997 - Character 29).
73. Pubis, length with respect to ischium length: shorter or equal (0); longer (1) (González Riga, 2003 - Character 33).
74. Metacarpals, distal phalangeal articular facets well developed: present (0); absent (1) (González Riga, 2003 - Character 32).
75. Ischium, posterior process twice or more the length of pubis articulation: present (0); absent (1) (Calvo *et al*., 2007a - Character 57).
76. Ischium, iliac pedicel: short and poorly developed (0); slender and well developed (1); wide and well developed (2) (Gonzalez Riga and Ortiz David, 2014 – Character 73).
77. Shape of preacetabular lobe of ilium: moderately expanded (0); broadly expanded and dorsally directed (1) (Gonzalez Riga and Ortiz David, 2014 – Character 74).
78. Orientation of preacetabular lobe of ilium: nearly vertical (0); nearly horizontal and outwardly projected (1) (Gonzalez Riga and Ortiz David, 2014 – Character 75).
79. Relative orientation of the pubic peduncle of ilium: angled (0); perpendicular with respect to the sacral axis (1) (Salgado *et al*., 1997 - Character 18).
80. Humerus/ femoral ratio of 0.90 o more: absent (0); present (1) (McIntosh 1990).
81. Lateral bulge of femur, below the major trochanter: absent (0); present (McIntosh 1990).
82. Proportion of distal end of tibia: more transversely than anteroposteriorly expanded (0); anteroposteriorly diameter of distal end of tibia subequal/greater than distal width (1) (Salgado *et al*., 1997 - Character 7).
83. Metatarsal I, length: shortest metatarsal (0); metatarsal V shorter than metatarsal I (1) (Curry Rogers, 2005 - Character 353).
84. Calcaneum: present (0); ossified calcaneum absent (1) (McIntosh 1990).
85. Osteoderm: absent (0); present (1) (Curry Rogers, 2005 - Character 363).
86. **Prezygapophyseal tuberosity on the lateral margin of the prezygapophysis of caudals 1-4: absent (0); present (1). (New).**
87. **Developed dorsal tuberosity on the sides of the base of the neural arch, above the transverse processes, that turns into a lateral ridge in the middle and posterior caudals: absent (0); present (1) (New).**
88. **Lamina suprapleurocoel: absent (0), present (1)**

**Table A. Character matrix.**

| Taxa  *Camarasaurus*  *Giraffatitan*  *Chubutisaurus*  *Andesaurus*  *Ligabuesaurus*  *Malawisaurus*  *Rukwatitan*  *Quetecsaurus*  *Mendozasaurus*  *Futalognkosaurus*  *Austroposeidon*  *Puertasaurus*  *Dreadnougthus*  *Isisaurus*  *Bonitasauria*  *Pellegrinisaurus*  *Epachthosaurus*  *Rapetosaurus*  *Ampelosaurus*  *Lirainosaurus*  *Rinconsaurus*  *Muyelensaurus*  *Gondwanatitan*  *Aeolosaurus colhuehuapensis*  *Aeolosaurus maximus*  *Aeolosaurus rionegrinus*  *Overosaurus*  *Opisthocoelicaudia*  *Alamosaurus*  *Neuquensaurus*  *Saltasaurus*  *Rocasaurus*  *Maxakalisaurus*  *Tapuiasaurus*  *Trigonosaurus*  *Uberabatitan*  *Drusilasauria*  *Baurutitan*  *Brasilotitan* | 1-10 11-20 21-30 31-40 41-50 51-60 61-70 71-80 81-88  0011000001 1001000000 0000000000 0000000000 0000000000 0000000000 0000000000 0000000000 0000000?  0001000011 0000000010 1011000000 0101000000 0000000000 0000000001 0000000000 0000001011 10000000  ?????????? ?????????? ?????????? ??????0000 11???01?0? ?00100?0?1 ?00???01?? ???0?????1 11???00?  ?????????? ?????????? ?????????? ?????31000 10?0111?0? 000?000001 10000?01?? ??1110???0 1????00?  ?????????? ?????????? 101?101?00 0201011100 1101?11??? ?????????? ?????100?? ?0?0?????1 11?????0  0??????11? ?0?????000 2011100110 0011131111 11??111??1 000?2000?1 1100???111 1?1?1????? ?1??1000  ?????????? ?????????? ????1?0??0 ??1??????1 1????????? 0?002000?1 1100???111 1???1????? ?1???00?  ???000???? ?????????? 221?100??0 0111?21100 1?00?????? ?000?????1 ??0??1?2?1 11?1?????? ??1??000  ?????????? ?????????? ????101??1 1222?31100 1200????11 0000210011 1100010111 1??1?????0 111?1000  ?????????? ?????????? ???2101011 0122231100 1100???111 ?0?0????11 ??0??????? ??1?12111? ?????000  ?????????? ?????????? ????1?0??1 0?1??31?00 1????????? ?????????? ?????????? ?????????? ???????1  ?????????? ?????????? ????1?0??0 0212?31100 1200?????? ???0?????? ?????????? ?????????? ???????0  ?????????? ?????????? 221?100??0 ?011?21101 101?11?111 ?000???111 0100010211 ??1?12111? ?????000  ?????????? ?????????? ????100??0 1?22?31100 1000????11 0?1022?000 100101001? ?01?11111? ?????000  ?11???111? 1?1??????1 221?100??0 1011?31111 1010111?11 0000320011 01001?01?1 ??1112???0 11???000  ?????????? ?????????? ?????????? ????230101 10????1110 0010320001 1100010111 ??11?11110 11110000  ?????????? ?????????? ?????????? ???????1?? ??1?11?110 ?210220001 20?0?????? ?????????? ?????100  111?001110 1111111110 2212100100 1011231111 10101111?0 0010320011 0?00010111 00?1101110 1?1?1000  ????0????? ?????????? 111?1011?0 ?12??21?11 101?11??11 ?000320001 01000?01?1 01???????? 1???1010  ?????????? ????????0? 221??????0 ?????31111 11??111?11 ?010320001 01000?01?1 01???????? 1???001?  ?????????? ??????????211?0101?0 1011?31111 11111111?? ?000320011 0101110111 0111111110 1????000  ?1????1??1 1???1?111? 211?0101?0 1011?31111 11111111?0 ?000320011 0101110111 0?11111110 11???000  ?????????? ?????????? ????1????1 ?????31111 111111111? ??11320102 02001?0??? ????0111?? ?1???00?  ?????????? ?????????? ?????????? ?????????? ?????????0 ?011320112 220??????? ?????????? ?????00?  ?????????? ?????????? ????0?0??? 1?11??1111 11110??1?0 ?01022?011 01001?01?? ??1??????? ?1???00?  ?????????? ?????????? 221??????? ?????3???? ????????30 ?011320112 02001?01?1 0?1101???? 11??100?    ?????????? ?????????? ????120??1 1011231111 1111111110 001122?0?1 1100?????? ??????111? ?????000  ?????????? ?????????? ?????????? 10??131111 0011100121 1000000021 0000011111 0111111110 1111000?  ?????????? ?????????? 22??000?00 1011?31111 11??000131 1000320021 0000010111 1111121110 11???000  ?????????? ?????????? ????011010 1011?31?11 111111?131 ?100321021 0000010111 01?112111? 11??1000  ?11?????11 1???01011? 221?020100 1011?31111 1111111110 ?100321021 0010011211 0111121110 11??1000  ?????????? ?????????? ????0????0 1?11?31111 111?111??? ?1003210?1 00100????? ??1?12111? 1????000  ?????????? ?????????? 221?000??0 1011??1?11 1?1??1???? ?1102111?? 1??010?1?? 1??1?????? ????1000  1011010?1? 1111????00 221?0????0 ?011231111 11111??11? ??1????1?? ?????01??1 ?1???????? ?????000  ?????????? ?????????? ????020??0 ?01?231111 101?11?0?0 001032?102 110000?111 11?????111 ?????000  ?????????? ?????????? ????110110 1011111?11 110111?000 1??10??011 0010?1???1 ?????????? ?????000  ?????????? ?????????? ?????????? ???1?31000 100?11???1 ?01?22001? 0?00000??? ?????????? ?????11?  ?????????? ?????????? ?????????? ?????????? ????????01 11?0200011 01010????? ?????????? ?????11?  ?????????? ?????????1 ?2??1101?? 101??????? ?????????? ?????????? ?????????? ?????????? ???????? |
| --- | --- |
